# Groundwater depletion in California’s Central Valley accelerates during megadrought

**DOI:** 10.1038/s41467-022-35582-x

**Published:** 2022-12-19

**Authors:** Pang-Wei Liu, James S. Famiglietti, Adam J. Purdy, Kyra H. Adams, Avery L. McEvoy, John T. Reager, Rajat Bindlish, David N. Wiese, Cédric H. David, Matthew Rodell

**Affiliations:** 1 Hydrological Sciences Laboratory, NASA Goddard Space Flight Center, Greenbelt, MD USA; 2 Science Systems and Applications, Inc, Lanham, MD USA; 3 Global Institute for Water Security, University of Saskatchewan, Saskatoon, SK Canada; 4 School of Environment and Sustainability, University of Saskatchewan, Saskatoon, SK Canada; 5 School of Sustainability, Arizona State University, Tempe, AZ USA; 6 Department of Applied Environmental Sciences, California State University Monterey Bay, Seaside, CA USA; 7 Jet Propulsion Laboratory, California Institute of Technology, Pasadena, CA USA; 8 Earth Science Division, NASA Goddard Space Flight Center, Greenbelt, MD USA; 9 Present Address: Rocky Mountain Institute, San Francisco, CA USA

**Keywords:** Hydrology, Water resources, Hydrology

## Abstract

Groundwater provides nearly half of irrigation water supply, and it enables resilience during drought, but in many regions of the world, it remains poorly, if at all managed. In heavily agricultural regions like California’s Central Valley, where groundwater management is being slowly implemented over a 27-year period that began in 2015, groundwater provides two–thirds or more of irrigation water during drought, which has led to falling water tables, drying wells, subsiding land, and its long-term disappearance. Here we use nearly two decades of observations from NASA’s GRACE satellite missions and show that the rate of groundwater depletion in the Central Valley has been accelerating since 2003 (1.86 km^3^/yr, 1961–2021; 2.41 km^3^/yr, 2003–2021; 8.58 km^3^/yr, 2019–2021), a period of megadrought in southwestern North America. Results suggest the need for expedited implementation of groundwater management in the Central Valley to ensure its availability during the increasingly intense droughts of the future.

## Abstract (web summary)

Liu et al. used the NASA GRACE/FO missions to show that since 2019, groundwater depletion in California’s Central Valley has accelerated by 31% compared to recent droughts, and has increased by a nearly a factor of 5 compared to the 60-year average.

## Introduction

Groundwater is a critical component of freshwater supplies for human life, for ecosystem and hydrological processes, for agricultural production, and more^1^. Groundwater is the major water source for roughly a third of the global population, and it supplies nearly half of the water used for irrigation^2^. However, groundwater resources have been under considerable stress around the world^2–6^, including in the arid and semi-arid western United States, where effective groundwater management is essential for sustainability^7^, yet where groundwater overuse is common^7,8^, and where climate change and changing hydrologic extremes are reducing opportunities for aquifer replenishment^9,10^.

Among the western states, California is the most populated, as well as the most productive agricultural region, both of which place heavy demands on freshwater resources. During the California droughts of the past two decades, surface water supplies decreased significantly, resulting in an increased reliance on groundwater pumping^11–13^. Groundwater supplies roughly two–thirds of California’s water supply during droughts, compared to one-third in non-drought conditions^12,14^. Drought-inducing weather patterns have been observed more frequently in recent years^15^, while the last two decades correspond to a period of megadrought in southwestern North America^16^. Although there is no strict definition of megadrought, in North America it typically refers to continued drought conditions lasting for longer than a decade^16^. These climatic changes have the potential to greatly intensify the stress on groundwater resources. Since groundwater use and depletion increase during drought, and natural groundwater recharge decreases, it also acts as a positive feedback to regional drying, with the potential to significantly worsen of the impacts of continued drought.

**Fig. 1:**
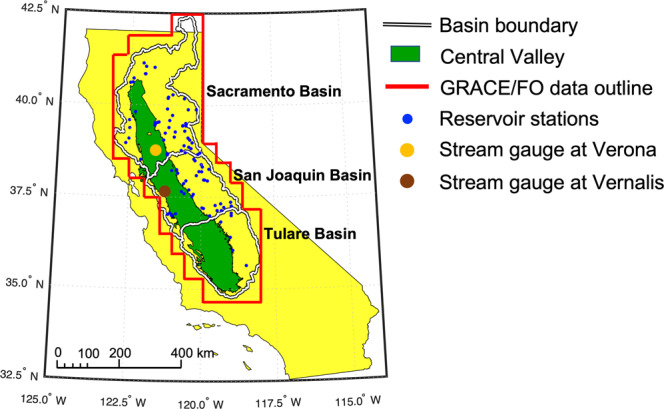
California’s Central Valley. The Central Valley (green) encompasses the Sacramento, San Joaquin, and Tulare Basins (black and white boundary). The red border outlines the area of GRACE/FO mascon data used for the study. Blue dots show locations of active reservoir storage gauges distributed within the study region, and the orange and brown dots show locations of the two main stream discharge gauges in Central Valley. The GRACE/FO data, reservoir storage and streamflow measurements are used to estimate groundwater storage changes as discussed in the Methods section.

California’s Central Valley is shown in Fig. 1. The region encompasses the Sacramento, San Joaquin, and Tulare basins, the major water sources for which are the mountain snowpack of the Sierra Nevada range. As one of the most important agricultural regions in the U.S., the Central Valley supplies 25% of the food consumed by the nation, with an estimated value of $17 billion per year, or 8% of the U. S. agricultural output by value^11^. However, widespread irrigated agriculture and a significant increase in permanent crops, such as vineyards and orchards, make it a region of extremely high groundwater consumption: the Central Valley is the second most-pumped groundwater aquifer system in U.S.^13^.

In addition to a shortage of renewable freshwater, overpumping groundwater has led to falling to water tables^17^, streamflow depletion^18^, declining water quality and wells running dry^2^, as well as to environmental hazards such as land subsidence^19,20^, and wastewater intrusion^21^. To combat these threats, California enacted its Sustainable Groundwater Management Act (SGMA) in 2014, requiring high- and medium-priority groundwater basins (as identified based on groundwater demands by the state’s SGMA Basin Prioritization^22^) to develop and implement management plans to achieve sustainable levels of groundwater pumping and recharge by 2042^23,24^. Among of these basins, nearly all of the groundwater basins located within Central Valley are classified as high- and medium priority, and nearly half are further designated critically overdrafted^22^. In response to SGMA, or to any groundwater management plan, it is essential to accurately monitor and characterize groundwater storage variations^25^.

Traditional in situ water table depth measurements from wells are the most direct approach to monitoring groundwater levels^2,7,23^. Groundwater monitoring wells are also used to characterize aquifer architecture and hydrogeological parameters, which are essential for building predictive models that support management decisions, including estimating groundwater availability^26^. However, it can be challenging to construct an accurate picture of groundwater levels from well data scattered across a regional domain due to the lack of a high density and fairly uniform distribution of monitoring wells. In addition, in the Central Valley, many farmers oppose expanding well monitoring under SGMA^25^. Therefore, it remains difficult to compile and standardize long term groundwater information at the regional scale through monitoring wells alone^23^.

To better characterize groundwater storage variations at the larger regional scales of aquifers, watersheds, states, etc., complementary studies have been conducted using either hydrologic modeling^11,13,24,27–29^ or remotely sensed observations^12,20,29–34^. The development of comprehensive groundwater models that consider human activities such as groundwater pumping, irrigation, farm practices, land subsidence, and other key processes, e.g. the Central Valley Hydrologic Model (CVHM), developed by the U. S. Geological Survey (USGS)^11,13^, is a major advance in modeling aquifer-scale groundwater behavior. Like most surface and groundwater models, although extremely important for research and water management applications, they require large amounts of monitoring well observations for model calibration and implementation, which is a labor-intensive, expensive and time-consuming process.

Studies using remotely sensing observations have demonstrated the proficiency of satellites for providing complementary information on groundwater variations in an efficient manner^29,35^, often over very large areas. For example, Liu et al. (2019) and Neely et al., (2021)^20,34^ observed land surface subsidence using Interferometric Synthetic Aperture Radar (InSAR) in high groundwater demand areas. Earth surface deformation in those regions was attributed and correlated to changes of groundwater storage. This technique requires long term, accumulated geodetic and groundwater observations to identify deformation due to groundwater changes and other factors, i.e. inelastic or elastic behavior, which are only available in certain data rich areas^29^.

The Gravity Recovery and Climate Experiment (GRACE) satellites, and its follow-on (GRACE-FO) mission, accurately and routinely measure earth’s time-variable gravity field, which is dominated by the redistribution of water over the globe^36^. Since the first GRACE mission was launched in 2002, it has allowed for tracking variations in total water storage (TWS; i.e. all of the snow, surface water, soil moisture and groundwater combined) at monthly and longer timescales, for regions that are 150,000 km^2^ or larger^37^, rather than local scales. The groundwater component of TWS can be isolated using a water storage balance approach^38^ by incorporating other hydrological measurements and estimates for snow water equivalent (SWE), and surface water and soil moisture storage. GRACE and GRACE-FO, hereafter referred to GRACE/FO, have been widely used to estimate groundwater storage changes^12,30–32,39,40^ and to monitor hydrological drought^33,41,42^. While the GRACE/FO satellite-based approach lacks the spatial and temporal detail of hydrological models and monitoring wells, it generally corresponds well with model simulations and in situ water balance and groundwater observations, and provides a reliable large-scale view of groundwater variations that is otherwise difficult to construct^23^. Famiglietti et al. (2011)^12^ integrated TWS anomalies measured from GRACE with estimates of SWE, soil moisture, and in situ observations of surface water storage to quantify groundwater storage variations in the Central Valley from 2003–2010. That study^12^ clearly identified the well-known California drought of 2006–2010, and provided the first space-based estimate of groundwater loss from the Central Valley.

In this work, we extend and update the previous study^12^ by using the nearly two decades of GRACE/FO observations to understand the longer-term variations of Central Valley groundwater, including their response to changing extremes of wet and dry periods, water management, and to the knowledge that SGMA will be entering its implementation phase within the next few years. Specific objectives of this study are to (1) retrospectively quantify phases of groundwater recharge and loss by integrating GRACE/FO-derived TWS with other terrestrial water components for the past two decades; (2) examine GRACE/FO-derived groundwater storage changes in the context of longer-term decadal trends and observations; (3) better understand the relationship between surface water allocations by the State of California and the U. S. federal government to estimated groundwater storage changes; and (4) to demonstrate the capability of GRACE/FO-derived groundwater storage changes to support regional groundwater management efforts. While this study is focused on the larger Central Valley and its Sacramento, San Jacinto, and Tulare sub-basins, and therefore may have limited impact on local-scale groundwater sustainability plans, as in previous studies^12,28,30^ it will provide a critical, ‘big-picture’ view of Central Valley-wide groundwater storage changes that may not otherwise be available from models and in situ well observations. This large-scale view has significantly raised awareness of the scope and urgency of ongoing groundwater depletion amongst decision makers and the general public, providing the critical understanding required for proposing and supporting improved groundwater management.

## Results

### Groundwater storage variations by integrating GRACE/FO-derived TWS with other terrestrial water storage components for the past two decades

**Fig. 2:**
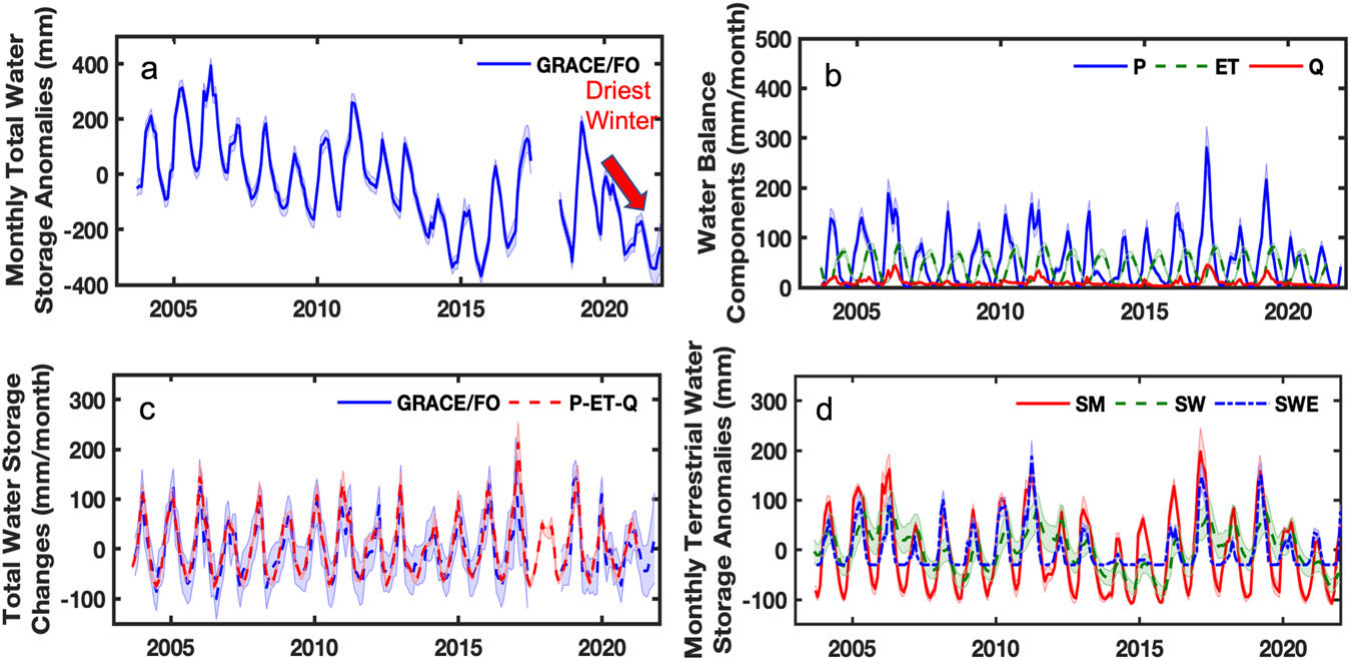
Datasets used for groundwater storage anomaly calculation and GRACE/FO data evaluation in the Central Valley. **a** GRACE/FO observed monthly total water storage (TWS) anomalies. Red arrow indicates the driest winter in TWS for the past two decades at the begining of 2021. **b** Three water balance fluxes of precipitation (P), evapotranspiration (ET), and streamflow (Q). **c** Comparison of monthly change in TWS (dS/dt) between that derived from GRACE/FO and from an observed water balance. **d** Anomalies of three TWS components of soil moisture (SM), surface water (SW), and snow water equivalent (SWE). All variables are represented in equivalent water height in millimeters for the study region.

GRACE/FO TWS anomalies for the combined Sacramento, San Joaquin and Tulare basins (Fig. 1, Fig. 2a) were used to calculate groundwater storage anomalies in California’s Central Valley. The GRACE/FO time series (Fig. 2a) for the combined basins is indicative of a region that has experienced successive droughts, punctuated by brief wet periods, resulting in significant cumulative water loss during the study period.

Before estimating groundwater storage changes, GRACE/FO TWS were first evaluated by comparing its monthly changes to those from an observed water balance calculation (see Eq.(1) in Methods). Figure 2b shows the observed water flux components including precipitation (P), evapotranspiration (ET) and streamflow discharge (Q) for the combined river basins, while Fig. 2c shows a close correspondence between dS/dt derived from GRACE/FO, and that computed using P–ET–Q in Eq.(1). The Root Mean Squared Difference between the two is 26.4 mm/month, and is within the range of the mean uncertainty using GRACE/FO measurements (43.6 mm/month). Such a good agreement between GRACE/FO-derived and observed dS/dt demonstrates that GRACE/FO is capable of accurately monitoring basin-wide water balance changes, and provides further confidence in the groundwater storage change estimates described below^12^.

Groundwater storage anomalies were estimated by subtracting the anomalies of soil moisture, surface water, and SWE (Fig. 2d) from GRACE/FO TWS anomalies (Fig. 2a) following Eq.(2) as detailed in Methods. The SWE, soil moisture and surface water datasets were obtained from operational, publicly available sources, including the National Oceanic Atmosphere Administration’s Snow Data Assimilation System (SNODAS)^43^, NASA’s North American Land Data Assimilation System (NLDAS)^44^, and the California Data Exchange Center^45^, respectively, ensuring data accessibility for potential routine monitoring following this approach.

**Fig. 3:**
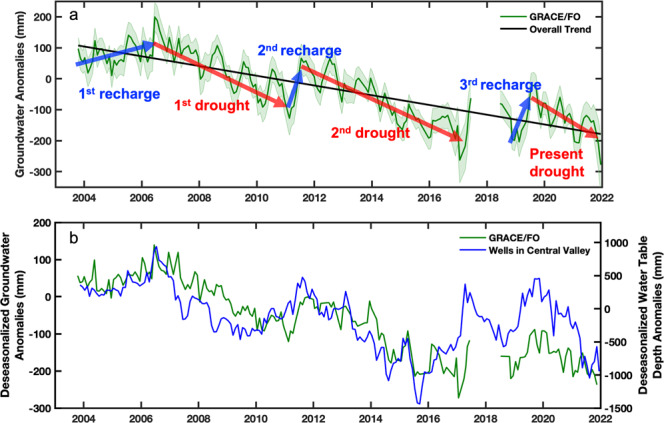
Groundwater storage variations in California’s Central Valley. **a** GRACE/FO-derived groundwater storage anomalies from September 2003–December 2021 in the Central Valley. The green shaded margin is the uncertainty of groundwater storage. Red arrows represent groundwater loss trends during the droughts of 2006–2011, 2011–2017, and since 2019. Blue arrows represent the three short recharge periods. The black line shows the groundwater depletion trend from 2003–2021. **b** comparison of deseasonalized anomalies of GRACE/FO derived groundwater and water table depth anomalies from monitoring wells in the Central Valley.

Figure 3a shows the monthly groundwater storage anomalies derived from GRACE/FO and the datasets shown in Figs. 2a, d in the Central Valley between September 2003 and December 2021. Three notable periods of groundwater recharge and loss were identified in the past 18 years. Groundwater recharge occurred during wet periods from October 2003 to July 2006, March 2011 to July 2011, and October 2018 to August 2019, shown as blue arrows in Fig. 3a. Groundwater loss phases correspond to the well-known droughts that occurred during that time period, namely August 2006–February 2011, August 2011–March 2017, and since September 2019, shown as red arrows in Fig. 3a. A pattern of short phases of recharge followed by longer phases of groundwater loss emerges, resulting in longer-term groundwater depletion over the last two decades. Estimated rates and the total volumes of groundwater gains and losses are summarized in Table 1.

**Table 1:** Groundwater change rates and total groundwater volume changes in the Central Valley

	Change rate (mm/yr)	Change rate (km^3^/yr)	Volume change (km^3^)
Oct. 2003–Jul. 2006 (1st recharge, Fig. 3a)	+22.7 ± 16.0	+3.49 ± 2.47	+9.9 ± 4.2
Aug. 2006–Feb. 2011 (1st drought, Fig. 3a)	−42.9 ± 7.8	−6.59 ± 1.20	−30.2 ± 2.6
Mar. 2011–Jul. 2011 (2nd recharge, Fig. 3a)	+462.5 ± 157.8	+71.07 ± 24.25	+29.6 ± 15.7
Aug. 2011–Mar. 2017 (2nd drought, Fig. 3a)	−42.7 ± 5.8	−6.56 ± 0.89	−37.2 ± 2.1
Oct. 2018–Aug. 2019 (3rd recharge, Fig. 3a)	+188.8 ± 108.9	+29.02 ± 16.73	+26.6 ± 16.0
Sep. 2019–Dec. 2021 (Present drought, Fig. 3a)	−55.8 ± 21.8	−8.58 ± 3.35	−20.0 ± 5.1
Sep. 2003–Aug. 2017 (Lifetime of GRACE)	−19.4 ± 2.1	−2.99 ± 0.33	−41.8 ± 1.2
Sep. 2003–Dec. 2021 (Megadrought, Fig. 3a)	−15.7 ± 1.4	−2.41 ± 0.22	−44.3 ± 0.9
1962–2021 (CVHM & GRACE/FO, Fig. 4)	−12.1 ± 0.8	−1.86 ± 0.12	−111.5 ± 0.9

The signs of – and + indicate groundwater losses and gains, respectively.

A groundwater recharge phase (22.7 ± 16.0 mm/yr; 3.49 ±  2.5 km^3^/yr) in the Central Valley was observed at the beginning of the GRACE mission during 2003–2006 (1st recharge in Fig. 3a and Table 1), when the precipitation amounts were close to or slightly higher than the 20-year average. The NOAA National Weather Service report^46^ reveals that weak to moderate levels of El Niño events during 2004–2006 resulted in nearly normal amounts of precipitation and snow in the study region. A volume of 9.9 ± 4.2 km^3^of groundwater was replenished during this phase of the analysis.

This period of groundwater increase was followed by the 4.5-year drought that began in August 2006. During the 2006–2011 drought (1st drought in Fig. 3a and Table 1), a groundwater loss rate of 42.9 ± 7.8 mm/yr (6.59 ± 1.20 km^3^/yr) was estimated, resulting in 30.2 ± 2.6 km^3^ of groundwater loss during that period. Compared with the earlier analysis in Ref. 12, an additional year of data was included here, and represented the complete drought phase through 2011, rather than through 2010, as in Ref. 12. Although the groundwater loss rate is slightly higher than the 38.9 ± 9.5 mm/yr reported in Ref. 12, the difference falls within the 95% confidence interval, confirming the consistency between the two analyses.

Prior to the second drought, a short, rapid recharge phase (March–July 2011, 2nd recharge in Fig. 3a and Table 1) replenished 29.6 ± 15.7 km^3^ of groundwater (462.5 ± 157.8 mm/yr; 71.07 ± 24.25 km^3^/yr), as a result of the strong El Niño in 2010 that brought abundant precipitation in early 2011^47^.

The groundwater loss rate for the second phase of drought in the GRACE/FO record (2011–2017, 2nd drought in Fig. 3a and Table 1) was 42.7 ± 5.8 mm/yr (6.56 ± 0.89 km^3^). Although a similar groundwater loss rate was estimated for the drought of 2006–2011, the second drought lasted a year longer, resulting in roughly 7 km^3^ more groundwater loss (37.1 ± 2.1 km^3^ total), equivalent to about 23% of surface water storage in the Central Valley, and greater than the volume of Lake Mead (32.2 km^3^) at full capacity. The GRACE/FO-based groundwater estimated in this study reached an 18-year low by late 2016. This phase of drought was notable for widespread water conservation efforts across California, and for the passage of SGMA in 2014. This second phase of drought ended with atmospheric river events that brought heavy precipitation to California in early 2017^48^.

The original GRACE mission was decommissioned in late 2017 and transitioned to GRACE-FO after its launch in May 2018. Hence there is year-long data gap in the combined GRACE/FO record from August 2017–September. 2018. Studies of that time period^23,34^ suggest that groundwater recharge occurred during this data gap. We estimate that during the lifetime of original GRACE mission (2003–2017), 41.8 ± 1.2 km^3^ of groundwater were lost (Table 1).

We assume that the groundwater depletion followed the 18-year historical trend (2003–2021), but made no assumption about its seasonal dynamics during the data gap between the GRACE and GRACE-FO missions. From October 2018 to August 2019 (3^rd^ recharge in Fig. 3a) we estimated that groundwater storage increased by 26.6 ± 16.0 km^3^ (188.8 ± 108.9 mm/yr; 29.02 ± 16.73 km^3^/yr).

The third phase of drought in the GRACE/FO record began in September 2019. After the recharge event in the winter of 2018, major water inputs in the region, including precipitation and SWE, significantly decreased in the winters of 2019 and 2020 (Figs. 2b and d). These two winters rank the years 2019 and 2020 as fourth driest consecutive 2-year period on record^49^. In particular, precipitation reached an 18 year low in the winter of 2020–2021 (Fig. 2b), and TWS (Fig. 2a) shows this same time period as the driest wet season in the GRACE/FO record. Between September 2019 and December 2021 (Present drought in Fig. 3a), total groundwater losses in the Central Valley were 20.0 ± 5.1 km^3^ (55.8 ± 21.8 mm/yr; 8.58 ± 3.35 km^3^/yr), which is roughly 31% faster than the previous two droughts.

During the present megadrought in southwestern North America (2003–2021), groundwater anomalies observed from GRACE/FO in the Central Valley show a trend of groundwater depletion of 15.7 ± 1.4 mm/yr (2.41 ± 0.22 km^3^/yr), resulting in a total groundwater loss of 44.3 ± 0.9 km^3^, an amount that is nearly than 1.4 times the full capacity of Lake Mead.

### Longer-term trends and comparison to observations

The GRACE/FO groundwater estimates were compared with water table depth anomalies observed from groundwater wells, as shown in Fig. 3b. A valley-wide water table depth was obtained by averaging measurements from available wells located within Central Valley, managed by California’s DWR and USGS^23^ (see Methods). Seasonal variations of GRACE/FO derived groundwater storage changes and the observed water table depth were removed by subtracting their climatologies, i.e. deseasonalized groundwater storage and water table anomalies, to avoid seasonal inconsistencies between the two measurements, and to only examine their long term trends. Overall, the two measurements demonstrate similar trends from 2003 to 2021. While there is a greater difference between the well and GRACE/FO estimates following 2017, Fig. 3b shows that the groundwater estimates using GRACE/FO are capable of capturing the periods of loss and recovery observed on the ground, and in particular, the greater rate of groundwater loss since 2019, which appears even stronger in the well observations than in the GRACE/FO estimates. Discrepancies may be attributed to the irregular availability of groundwater well data, and to a major decline in available well observations since late 2018 (see Methods, Supporting Information, and Fig. S3). Both of these factors underscore the challenges of estimating large-area groundwater dynamics from well data alone, and of validating groundwater models and satellite observations.

**Fig. 4:**
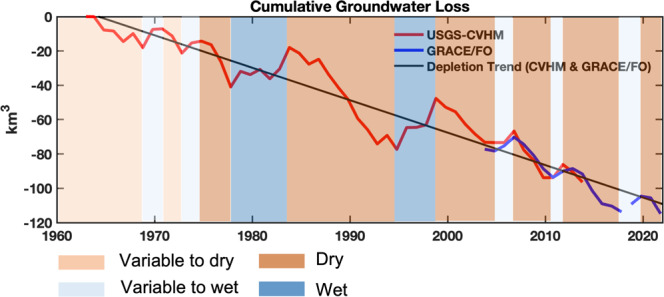
Yearly cumulative groundwater losses in the Central Valley. Groundwater losses combining the USGS’s Central Valley Hydrologic Model (CVHM)^13^ and the GRACE/FO estimates since 1962. The black line represents the overall groundwater depletion from 1962 to 2021 calculated by combining the CVHM and GRACE estimates.

Figure 4 shows cumulative groundwater losses from 1962–2021 using the CVHM^13^ and GRACE/FO. From 2003 to 2014 when both CVHM and GRACE data were available, the groundwater depletion rate for the CVHM was 16.3 ± 6.3 mm/yr (2.51 ± 0.97 km^3^), matching that from GRACE, 14.7 ± 6.0 mm/yr (2.25 ± 0.92 km^3^), indicating that the two methods are compatible and may be combined for the further analysis. The combined CVHM-GRACE/FO groundwater depletion rate was calculated by using both CVHM estimations from 1962–2014 and GRACE-derived groundwater storage changes from 2003–2021 through linear regression analysis. The result shows that the groundwater depletion rate from 1962 to 2021 was 12.1 ± 0.8 mm/yr (1.86 ± 0.12 km^3^/yr), shown as the black line in Fig. 4, resulting in a total groundwater loss of 111.5 ± 0.9 km^3^. In addition, Fig. 4 shows that the periods for groundwater recovery were shorter, and mostly driven by extreme weather events^46–48,50^ in the nearly two decades of the GRACE/FO record. Although groundwater was recharged, these extreme wet events typically generated flooding, and had significant negative social, environmental and economic consequences^46–48,50^. This sequence of extreme hydrological events—long-term extremely dry conditions with considerable groundwater losses, punctuated by short-term extremely wet conditions with short bursts of groundwater recharge—underscores the challenge of sustainable groundwater management under changing climate.

Figures 3a and 4, along with Table 1, show that the rate of groundwater loss is accelerating in the Central Valley. Groundwater loss rates observed from GRACE/FO (15.7 ± 1.4 mm/yr; 2.41 ± 0.22 km^3^/yr) between 2003 and 2021 are 28% faster than the longer-term (1962–2021) depletion rate of the combined CVHM-GRACE/FO record (12.1 ± 0.8 mm/yr; 1.86 ± 0.12 km^3^/yr). The most recent phase of groundwater loss, between September 2019 and August 2021 (55.8 ± 21.8 mm/yr; 8.58 ± 3.35 km^3^/yr), is nearly 31% faster than GRACE/FO estimated losses the previous two drought phases during the GRACE/FO record, and nearly five times faster than the long-term depletion rate.

### Relationship between surface water allocations and estimated groundwater storage changes

**Fig. 5:**
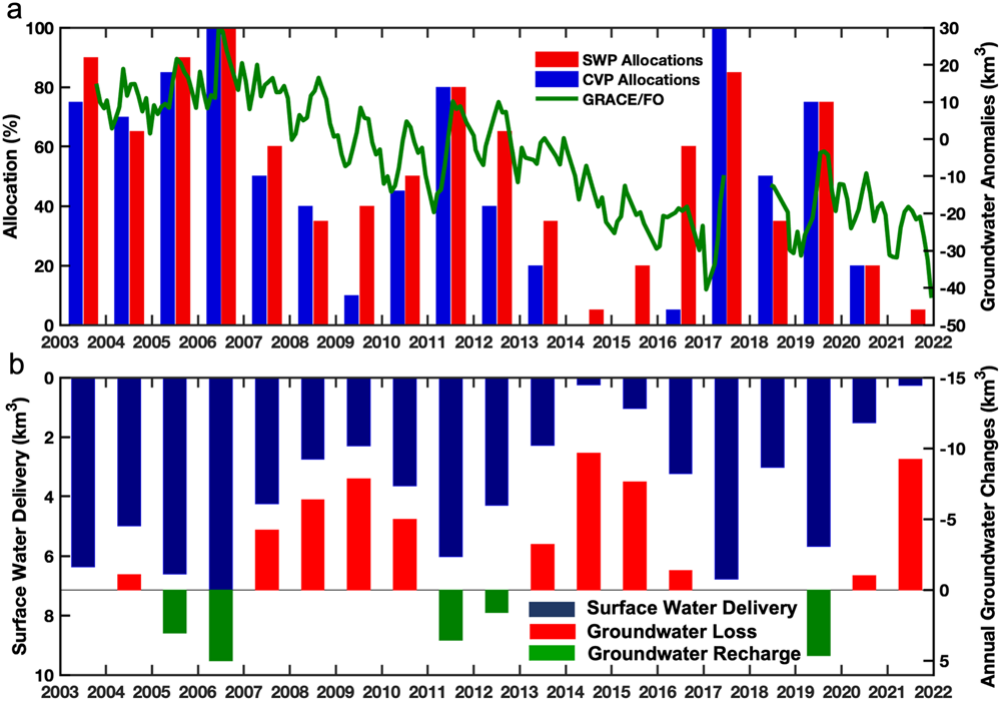
Groundwater and surface water management in Central Valley. **a** Comparison between annual surface water allocations in the aqueducts of the California State Water Project (SWP) and the federal Central Valley Water Project (CVP) and GRACE/FO-derived groundwater storage anomalies. **b** Comparison between annual surface water deliveries (dark blue bars) of SWP and CVP to the GRACE/FO derived groundwater changes (red and green bars) in Central Valley. The groundwater changes in 2003, 2017, and 2018 are not included because GRACE/FO-derived data do not have complete coverage over the year.

Figure 5a compares GRACE/FO estimated monthly groundwater storage variations to annual surface water allocations (in % of annual maximum) via the two primary aqueducts in the Central Valley, the California State Water Project (SWP)^51^ and the federal Central Valley Water Project (CVP)^52^. The two aqueducts transport surface water from northern California to the south. Figure 5b compares the annual groundwater storage changes (net fluxes) to the total surface water deliveries from both the CVP and SWP (in km^3^). The annual groundwater change was calculated as the difference of the mean annual groundwater anomalies between two consecutive years. Figure 5a, b show that when surface water is abundant, greater allocations are made to farmers, relieving stress on groundwater and allowing for recovery, and vice versa.

Between 2003 and 2007, surface water storage was increasing (Fig. 2d), allowing for larger allocations (>60%) from both aqueducts, less reliance on groundwater, and hence increasing groundwater storage. Surface water deliveries in Central Valley reached a high for the study period in 2016, resulting in about 5 km^3^ recharge (Fig. 5b). Surface water storage, and hence allocations, decreased between 2007 and 2009, resulting in significant groundwater storage decline. Surface water deliveries decreased to 2.30 km^3^ in 2009, corresponding to the highest annual groundwater storage loss by 7.86 km^3^ during the 1st drought period.

The second drought in the GRACE/FO record began in August 2011, triggering decreasing surface water allocations that resulted in heavy groundwater demand. During this period, CVP cut its allocation to 0% in 2014 and 2015, and 5% in 2016, while the SWP reached its lowest allocation for the study period, 5% in 2014. The low surface water delivery volumes in 2014 and 2015 drove corresponding annual groundwater losses of 9.66 and 7.64 km^3^, respectively, and led to intensified groundwater pumping through 2016 (Fig. 5b).

Groundwater storage variations continued to reflect surface water allocations, increasing in 2017 and 2019 with above-average surface water storage, followed by major losses in both surface water allocations, and groundwater storage, through the end of 2021. For example, in 2020, aqueduct allocations decreased to 20% for both projects, and to 0% and 5% in 2021 for the CVP and SWP, explaining in part the increased rate of groundwater loss during this time period. In 2021, the annual groundwater loss was 9.22 km^3^, matching the greatest annual loss during the study period, which occurred in 2014.

### Demonstration of GRACE/FO-derived groundwater storage changes to support regional groundwater management

**Fig. 6:**
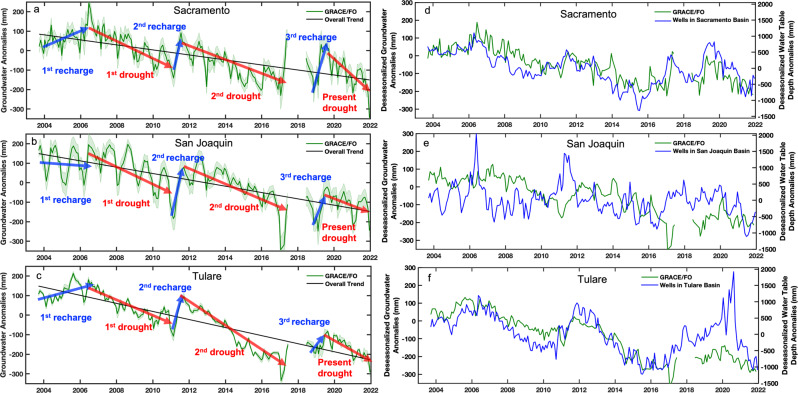
Groundwater storage variations in the three Central Valley sub-basins. GRACE/FO-derived groundwater anomalies during September 2003–December 2021 in the (**a**) Sacramento, (**b**) San Joaquin, and (**c**) Tulare basins. The green shaded margins are the uncertainty of groundwater storage estimates. Red arrows represent groundwater loss trends during the droughts of 2006–2011, 2011–2017, and since 2019. Blue arrows represent the three short recharge periods. The black line shows the overall groundwater depletion trend from 2003–2021. Comparison of deseasonalized anomalies of GRACE/FO derived groundwater and water table depth anomalies from monitoring wells for the (**d**) Sacramento, **e** San Joaquin, and (**f**) Tulare basins.

GRACE/FO-derived groundwater storage changes were also estimated in the Sacramento, San Joaquin, and Tulare basins, as shown in Fig. 6 and Table 2. The same periods of groundwater recharge and loss in the Central Valley are used to calculate the gains and losses for the three basins, including longer-term depletion rates. Overall, the individual basin follows similar trends, i.e. three short recharge phases, followed by three longer droughts, as was presented for the entire Central Valley. During the 1^st^ recharge phase, similar rates of groundwater recharge can be observed in the Sacramento and Tulare basins, with increasing rates of 39.0 ± 20.0 and 27.5 ± 15.8 mm/yr (2.81 ± 1.44 and 1.17 ± 0.67 km^3^/yr (Fig. 6a, c and Table 2)), resulting in groundwater increases of 8.0 ± 2.4 km^3^ and 3.3 ± 1.1 km^3^ in the two basins, respectively. Although a slight groundwater loss of 0.7 ± 2.0 km^3^ (6.4 ± 29.6 mm/yr; 0.26 ± 1.21 km^3^/yr) in the San Joaquin basin is observed for this period (Fig. 6b and Table 2), the loss rate is not statistically significant (within an uncertainty of 95% confidence interval), indicating that groundwater supply and consumption were nearly balanced in the basin.

**Table 2:** Groundwater change rates and total groundwater volume changes in the three sub-basins in the study region

	Sacramento (72,015 km^3^)			San Joaquin (40,986 km^3^)			Tulare (42,512 km^3^)		
	Change rate (mm/yr)	Change rate (km^3^/yr)	Volume change (km^3^)	Change rate (mm/yr)	Change rate (km^3^/yr)	Volume change (km^3^)	Change rate (mm/yr)	Change rate (km^3^/yr)	Volume change (km^3^)
Oct. 2003–Jul. 2006 (1st recharge, Fig. 6a–c)	+39.0 ± 20.0	+2.81 ± 1.44	+8.0 ± 2.4	−6.4 ± 29.6	−0.26 ± 1.21	−0.7 ± 2.0	+27.5 ± 15.8	+1.17 ± 0.67	+3.3 ± 1.1
Aug. 2006–Feb. 2011 (1st drought, Fig. 6a–c)	−43.7 ± 8.7	−3.15 ± 0.63	−14.4 ± 1.3	−44.7 ± 12.9	−1.83 ± 0.53	−8.4 ± 1.1	−40.1 ± 6.3	−1.70 ± 0.27	−7.8 ± 0.6
Mar. 2011–Jul. 2011 (2nd recharge, Fig. 6a–c)	+570.6 ± 117.2	+41.09 ± 8.44	+17.1 ± 5.4	+627.4 ± 135.1	+25.71 ± 5.54	+10.7 ± 3.6	+439.2 ± 68.7	+18.67 ± 2.92	+7.8 ± 1.9
Aug. 2011–Mar. 2017 (2nd drought, Fig. 6a–c)	−34.0 ± 8.0	−2.45 ± 0.58	−13.9 ± 1.4	−40.4 ± 9.9	−1.66 ± 0.41	−9.4 ± 1.0	−62.9 ± 4.4	−2.67 ± 0.19	−15.1 ± 0.4
Oct. 2018–Aug. 2019 (3^rd^ recharge, Fig. 6a–c)	+309.0 ± 123.6	+22.25 ± 8.90	+20.4 ± 8.5	+225.0 ± 123.7	+9.22 ± 5.07	+8.5 ± 4.9	+125.3 ± 64.3	+5.33 ± 2.73	+4.9 ± 2.6
Sep. 2019–Dec. 2021 (Present drought, Fig. 6a–c)	−76.1 ± 28.1	−5.48 ± 2.02	−12.8 ± 3.1	−38.1 ± 25.2	−1.56 ± 1.03	−3.6 ± 1.6	−60.1 ± 14.0	−2.55 ± 0.60	−6.0 ± 0.9
Sep. 2003–Aug. 2017 (Lifetime of GRACE)	−16.9 ± 2.6	−1.22 ± 0.18	−17.1 ± 0.7	−17.7 ± 3.2	−0.73 ± 0.13	−10.2 ± 0.5	−25.9 ± 2.3	−1.10 ± 0.10	−15.4 ± 0.4
Sep. 2003–Dec. 2021 (Megadrought, Fig. 6a–c)	−12.9 ± 1.8	−0.93 ± 0.13	−17.0 ± 0.5	−16.2 ± 1.9	−0.67 ± 0.08	-12.2 ± 0.3	−20.6 ± 1.5	-0.88 ± 0.06	−16.1 ± 0.3

The signs of – and + indicate groundwater losses and gains, respectively.

When entering to the 1st drought phase, results show that the Sacramento, San Joaquin, and Tulare basins all experienced similar groundwater loss rates of ~42 mm/yr (40–44 mm/yr) (Fig.6a–c and Table 2). The drought ended with the strong El Niño in 2010^47^.

During the 2^nd^ drought, all three basins experienced significant losing trends. Figure 6a–c, and Table 2 show that the Tulare basin suffered more severe groundwater losses than the other basins, with a loss rate of 62.9 ± 4.4 mm/yr (−2.67 ± 0.19 km^3^/yr). The total groundwater loss in the Tulare basin was 15.1 ± 0.4 km^3^, which was nearly 40% of the total loss in Central Valley, yet the area of the Tulare basin only occupies about one quarter of the study region. The groundwater storage changes during the 18 year study period show that the depletion rates in the Sacramento, San Joaquin, and Tulare basins, were 12.9 ± 1.8, 16.2 ± 1.9, and 20.6 ± 1.5 mm/yr (0.93 ± 0.13, 0.67 ± 0.08, and 0.88 ± 0.06 km^3^/yr) (Fig. 6a–c and Table 2), respectively, indicating that the southern Central Valley (combined San Joaquin and Tulare) lost more groundwater than the north, similar to the findings of earlier studies^23,30^. However, the situation was reversed in the drought that began in September 2019 (present drought in Fig. 6a–c), during which we found higher groundwater loss rates of 76.1 ± 28.1 mm/yr (5.48 ± 2.02 km^3^/yr) in the Sacramento basin compared to those of 38.1 ± 25.2 and 60.1 ± 14.0 mm/yr (1.56 ± 1.03 and 2.55 ± 0.60 km^3^/yr) for the San Joaquin and Tulare basins, respectively.

The deseasonalized GRACE/FO-derived groundwater storage and observed water table anomalies are compared for each of the three basins. Similar to the approach for the whole Central Valley, wells with available measurements within a particular basin boundary were averaged to represent the water table depth variation for the basin (see Methods and Supplementary Information). The two measurements show similar trends and variations for the Sacramento and Tulare basins, except for a strong water table rise in the winter of 2019 for the Tulare basin. As discussed earlier for the entire Central Valley, a dramatic decrease in the number of available well observations after late 2018 may have resulted in an inconsistent record of water table depth.

While the Sacramento and Tulare basins showed generally good agreement between GRACE/FO-derived groundwater storage changes and observed well measurements, less correspondence was observed in the San Joaquin basin, particularly during the 1st drought period. However, the two drought phases from 2011–2017 and after 2019 are clearly recognizable, with water table observations falling in response to increased groundwater pumping.

Figure 6 highlights both strengths and weaknesses of using the GRACE/FO approach at the sub-basin scale of the individual Sacramento, San Joaquin, and Tulare basins. On the one hand, sub-basin analyses provide important insights into groundwater storage variations across the Valley, in particular, sub-basin trends, which could ultimately inform SGMA performance and provide early warning (in the case of the Sacramento basin) for those regions where groundwater losses are unexpected. On the other, the sub-basins are considerably smaller than the ~154,000 km^2^ area of the Central Valley, which corresponds the lower area limit for an acceptable level of error for monthly TWSA detection^36,53–55^. (Note that the longer time period associated with the trend calculations mitigates this issue somewhat, resulting in greater confidence in the sub-basin trends than the monthly variations). Hence the GRACE/FO-derived groundwater storage variations at these sub-basin scales should be used judiciously.

As with the whole-valley comparisons to observations, the sub-basin analyses are faced with the same challenges as described above, i.e. the difficulties in assembling larger-area water table depth averages from unevenly distributed well observations collected at disparate times and for varying periods of time. In spite of these challenges, the regional groundwater analyses for the sub-basins demonstrates the potential utility of GRACE/FO-derived groundwater storage changes for supporting regional groundwater management efforts.

## Discussion

The current trajectory of groundwater storage in the Central Valley, shown in Figs. 3a and 6a–c since 2003, and over the last 6 decades in Fig. 4, shows a clear pattern of brief groundwater recovery events during shorter wet periods, followed by longer periods of groundwater loss during drought, and an overall trend of long-term groundwater depletion. This well-established pattern is largely driven by irrigation needs for agricultural production^11–13,30^. When annual surface water allocations delivered to farmers by the SWP and the CVP aqueducts are reduced, farmers have little choice but to use more groundwater. This is demonstrated in Fig. 5a–b.

The GRACE/FO missions have been operating during a period of megadrought in California and southwestern North America. The years 2000–2021 represent the driest 22-year period since at least 800, which may be a harbinger of more global warming-fueled extreme megadrought in the future^16^. Stress on groundwater resources under these drying conditions will likely increase in the coming decades^6^, and will be exacerbated by the need to provide more water and produce more food for a growing population. These suggest that the patterns of groundwater storage variations documented here are unlikely to change without significant groundwater management intervention.

Hence, the results reported here have important implications for the future of SGMA and water management in California, especially since shortage conditions have been declared for the first time in history on the Colorado River^56^, which will ultimately limit the allocation of surface water to California should these conditions persist or worsen. Some key findings from this study are: (1) groundwater loss rates in the Central Valley measured by GRACE/FO are accelerating over the past two decades, relative to the long-term depletion rate observed by combining the CVHM and GRACE/FO (1.86 km^3^/yr, 1961–2021; 2.41 km^3^/yr, 2003–2021; 8.58 km^3^/yr, 2019–2021) as listed in Table 1; (2) TWS from GRACE/FO showed that the wet season of calendar year 2021 (winter 2020–2021) was identified as the driest wet season of the study period, as shown in Fig. 2a; (3) wet/groundwater recharge periods during the last two decades were short as compared with drought/groundwater loss periods; and (4) that even the normally wetter, northern half of the Central Valley is now suffering from groundwater losses (Table 2). Taken together, these results underscore the importance of SGMA for groundwater management and of the GRACE/FO missions for providing basin- and valley-wide ‘big picture’^35^ assessments of the state of groundwater storage variations. GRACE-estimates of groundwater storage variations^12^ were noted by the California State Water Resources Control Board as being critical to raising awareness of the need for SGMA in 2014. The work presented here may well raise awareness of the need to accelerate its implementation to aggressively slow rates of groundwater depletion.

The impact of the present megadrought on groundwater storage is shown very clearly in Fig. 5. When SWP and CVP allocations and deliveries increase, groundwater storage recovers, and vice versa. In 12 of the 18 years studied here, one or both of the SWP and CVP surface water allocations fell below the 50% level, contributing to 1.4 Lake Mead’s worth of groundwater depletion. Since groundwater supplies are limited, and continued depletion is resulting in several negative consequences (falling water tables, drying wells, increasing pumping and well-drilling costs, decreasing groundwater access, land subsidence, declining groundwater quality, streamflow depletion^2^) continued overdrafting of groundwater supplies during drought is surely unsustainable in the long term, in particular in the face of their increasing frequency and severity. This underscores the importance of SGMA, while Fig. 5 highlights the urgent need for conjunctive management of surface and groundwater resources. Furthermore, since groundwater losses are accelerating rather than decelerating ahead of SGMA implementation, which may well be in anticipation of impending restrictions, results suggest the need for expedited implementation of groundwater management in the Central Valley to ensure its availability during the increasingly intense droughts of the future.

## Methods

### Total water storage from GRACE and GRACE-FO

Monthly estimates of total water storage (TWS) are taken from the JPL RL06M Version 2 GRACE/FO mascon solution^53,54^. Two post-processing algorithms are applied: the Coastal Resolution Improvement (CRI) filter to reduce land/ocean leakage errors, as well as gridded gain factors which act to redistribute mass (in a mass conserving fashion) at 0.5^o^ resolution within each 3^o^ mascon element^55^. The application of both algorithms allows for an exact averaging kernel to be applied when estimating mass over the study region. The TWS time series for the study region, shown in Fig. 2a, was extracted using a mascon boundary (Fig. 1) which covers the Sacramento, San Joaquin, and Tulare basins. Uncertainty of the TWS estimates was computed by accounting for both measurement and leakage errors^55^ and was provided in the GRACE/FO mascon dataset. This study used the data from September 2003, coincident with the starting date of the Snow Water Equivalent data, to December 2021.

A water balance approach was used to compare GRACE/FO-derived TWS change to observations, given as:

1 $${{{{{\rm{dS}}}}}}/{{{{{\rm{dt}}}}}}={{{{{\rm{P}}}}}}-{{{{{\rm{ET}}}}}}-{{{{{\rm{Q}}}}}}$$

where dS/dt is the change of total water storage in a given time *t* (monthly for the study), P is precipitation from the monthly PRISM 4-km product^57–59^, ET is monthly evapotranspiration derived from MODIS observations and MERRA-2 meteorological data using the Priestley Tylor-Jet Propulsion Laboratory (PT-JPL) model^59^, and Q is the surface water outflow from the Central Valley watershed. The surface water outflow in the region is primarily discharged from the Sacramento and San Joaquin rivers into the San Francisco Bay estuary through the Sacramento-San Joaquin Delta area^60^. In the study, we compiled streamflow measurements from the gauging stations at Verona and Vernalis, operated and maintained by the U.S. Geological Survey (USGS)^61^. These two gauges record stream discharge from the main streams of the Sacramento and San Joaquin rivers before reaching the Delta^28^, as shown in Fig. 1. The water fluxes of P, ET, and Q, are given as integrated monthly totals, and are expressed as basin-averaged depths (detailed in S1). Thus, dS/dt represents the change of total water storage each month. Figure 2b shows P, ET and Q for the study period and Fig. 2c shows the comparison of dS/dt from GRACE/FO and from the observed water balance approach.

### Estimating groundwater variations

Groundwater variations, computed as anomalies (GWA), were obtained by subtracting the terrestrial water mass anomalies in soil moisture (SMA), snow water equivalent (SWEA) and surface water storage (SWA) from the total water storage anomalies (TWSA) measured from GRACE/FO:

2 $${{{{{\rm{GWA}}}}}}={{{{{\rm{TWSA}}}}}} \, {-} \, {{{{{\rm{SMA}}}}}} \, {-} \, {{{{{\rm{SWEA}}}}}}-{{{{{\rm{SWA}}}}}}$$

In this study, SM in the 0–200 cm layer was obtained from the NLDAS phase 2 product (NLDAS-2) over the study region^44^. The NLDAS-2 provides monthly SM estimates from three land surface models, including Noah^62^, Mosaic^63^, and the Variable Infiltration Capacity (VIC) model^64^, at a spatial resolution of 0.125^o^. Soil moisture is recorded in kg/m^2^ and was converted into equivalent water height in millimeter (EWH in mm), as detailed in S2. The mean SM of the three models was used for the groundwater calculation. The uncertainty in the mean SM was estimated from the standard deviation of the three models. The NLDAS-2 product has been fully assessed for its applicability^65,66^.

The monthly SW storage was obtained from 92 in situ gauges of dams and reservoirs^45^, managed by California’s Department of Water Resources (DWR), in the study region (Fig. 1). These active gauges provide monthly mean reservoir storages in volume in units of acre-feet. Measurements from these gauges were compiled and summed to estimate the majority of the surface water storage in the study region. The units of acre-feet were then converted into EWH in mm using the area of the basin, as shown in S2. Uncertainty of SW was set at 15% of the SW storage since no published error estimates for these gauges are available^12^.

The SWE was obtained from the SNODAS data product^43^, which was estimated by fusing the remotely sensed estimates and in situ observations assimilated into a Snow Thermal Model (SNTHERM.89) by the National Operational Hydrologic Remote Sensing Center. SNODAS provides daily SWE estimates at a spatial resolution of 1 km. Monthly SWE was obtained by averaging the daily measurements to the monthly time scale. The accuracy of SNODAS SWE has been reported from ~10–20% of the SWE values at the basin scale for different regions^67–69^. Therefore, we assume an uncertainty of 15% for SWE.

These terrestrial water storage components were aggregated into the Central Valley and individual basin domains and transformed to anomalies by subtracting their historical means for groundwater variation calculation. Anomalies of these components are shown in Fig. 2d and Fig. S1 for the Central Valley and individual basins, respectively. For groundwater calculations in the study region (Eq. 2), we assumed that the mountains surrounding the basins contain limited capacity for groundwater storage^12^, though Argus et al. (2017)^70^ present evidence for some mountain storage. Following Famiglietti et al., (2011)^12^, we attribute the groundwater variations derived from GRACE/FO as having occurred in the Central Valley and each basin.

### Estimating groundwater recharges and depletion

We identified three phases of groundwater recharge, from October 2003 to July 2006, from March 2011 to July 2011, and October 2018 to July 2019. We also identified groundwater loss phases during the two notable California droughts of August 2006 to February 2011 and August 2011 to March 2017, and the ongoing drought since 2019. Trends for recharge and loss phases were calculated by linear regression for each period, and a student’s-t test was applied with a confidence interval of 95% to estimate trend uncertainty (detailed in S3), as shown in Fig. 3a and Table 1. The groundwater change rates from equivalent water depth in (mm/yr) were converted to volume change (km^3^/yr) by times the area of study region. Finally, the total amount of groundwater recharge or loss was calculated by multiplying by the length of recharge or drought period. The same process was applied to Sacramento, San Joaquin, and Tulare basins for regional analysis, (Fig. 6 and Table 2).

### Auxiliary datasets

In situ well measurements within the Central Valley were used to compare observed groundwater variations with those estimated here. The well data were processed using original datasets from groundwater monitoring networks managed by California’s DWR and the USGS^23^. A quality control process was applied to remove erroneous recordings and missing data to ensure uniformity in each station^23^, detailed in Supporting Information (S4). Averaged water table depth below the surface from >1000 wells across the study region (Fig. S2) was calculated in order to best represent groundwater variations across the area for comparison. Although the spatial and temporal distribution of available wells across the Central Valley is highly variable over time (as shown in Fig. S2 and S3), the well data provide valuable information to support the use of GRACE/FO signal to understand groundwater storage changes. The water table depth and GRCAE/FO derived groundwater anomalies were deseasonalized (see S5) to remove the potential phase mismatch between the two datasets for the comparison as discussed in the Results section.

Historical (1962–2014) cumulative groundwater loses were obtained from the USGS Central Valley Hydrology model (CVHM)^11,13^. The CVHM is a comprehensively calibrated hydrology model using in situ monitoring wells and is developed to provide water resource information to decision makers who are engaged in managing the Central Valley aquifer system. In this study, annually averaged data from the CVHM and GRACE/FO-derived groundwater storage were used for the comparison. The GRACE/FO-derived cumulative change is placed into historical context by removing a bias using concurrent data (2003–2014). Then, the historical groundwater loss from 1962–2021 is quantified by using both estimates through linear regression (Table 1).

Finally, the surface water allocation in percentage and delivery amounts, determined by the federal CVP and the California SWP^51,52^, and distributed in their aqueducts, were used to understand how the variations in surface water availability drive farmers’ groundwater usage patterns (Fig. 5).

## Supplementary information

Supplementary Information

## Data Availability

Datasets of GRACE/FO, NLDAS-2, SNODAS, surface water storage, PRISM precipitation, USGS stream discharge, and surface water allocations of the CVP and SWP are publicly available and their archived data portals are provided in the Reference section. The monitoring well measurements and evapotranspiration estimates, are supported by co-authors Dr. Kyra H. Adams at NASA Jet Propulsion Laboratory/Caltech and Dr. A. J. Purdy at California State University at Monterey Bay. These raw data will be available upon request. All processed datasets aggregated to the Central Valley and the three individual basin domains have been deposited in Zenodo at https://zenodo.org/record/7392554 (10.5281/zenodo.7392554).

## Code availability

The codes necessary to reproduce our results have been deposited in Zenodo at https://zenodo.org/record/7392560 (10.5281/zenodo.7392560).
