# Supplementary material for: Groundwater depletion in California’s Central Valley accelerates during megadrought
Source: Nat Commun. 2022 Dec 19;13:7825. doi: 10.1038/s41467-022-35582-x (PMC9763392; doi:10.1038/s41467-022-35582-x)
Supplement: Supplementary file 1 — Supplementary Information [file 41467_2022_35582_MOESM1_ESM.pdf]

Supplementary Materials for

Groundwater Depletion in California's Central Valley Accelerates During Megadrought

Pang-Wei Liu, James S. Famiglietti*, Adam J. Purdy, Kyra H. Kim, Avery L. McEvoy, John T. Reager, Rajat Bindlish, David N. Wiese, Cedric H. David, and Matthew Rodell

*Corresponding author. Email: jay.famiglietti@usask.ca

This file includes:

Supplementary Information
Sections S1-S5
Figs. S1 and S5

Supplementary Information

We provide information on data processing for water fluxes **(S1)**, terrestrial water components **(S2)**, groundwater depletion estimation and its uncertainty estimation **(S3)**, in situ well measurements **(S4)**, and deseasonalization processing **(S5)**. **Figure S1** presents the terrestrial water storage components in the three basins. **Figures S2 and S3** show the in situ well distribution map and their measurements aggregated into the Central Valley and the three sub-basins in the study area. **Figure S4 and S5** present the deseasonalization steps for well measurements and groundwater estimates.

S1. Water fluxes for water balance analysis

The water flux components including precipitation (P), evapotranspiration (ET), and streamflow discharge (Q) from PRISM, PT-JPL, and USGS gauges were integrated to monthly totals and expressed as basin-averaged depths in millimeters (mm) for water balance analysis. The PRISM and PT-JPL datasets originally record P and ET as monthly cumulative values in mm, so their time series data were extracted by averaging values over the domain as shown in Fig 1. The Q values from USGS gauges were recorded in cubit-feet per second on a daily basis (Q_{cfs}). To convert Q_{cfs} to monthly values in mm (Q_{mm}), the equation (S1) is used:

$$Q_{mm} = \sum_{d=1}^{n} \frac{Q_{cfs,d} \times q_1}{A} \times 10^6 * 3600 * 24 \quad \text{(S1)}$$

where, d represents the dth day of the month and n is total days of the month. q_1 is a conversion factor, $2.83168 \text{x} 10^{-11}$, for cubit-feet to km^3, and A is the area of the study region, equivalent to 153,659 km^2. The P, ET, and Q values are presented in Fig. 2(B) and used to calculate the water balance using equation (1) as shown in the Fig. 2(C) in the main text.

S2. Surface water components for groundwater storage change estimation

The monthly soil moisture data from NLDAS is recorded in kg/m^2. Using conventional water density, i.e. 1000 kg/m^2, the SM value of NLDAS is equivalent to Equivalent Water Height in millimeters (EWH, mm).

The surface water storage from *in situ* gauges of dams and reservoirs are recorded in units of acre-feet (SW_{AF}). In the study, surface water storage (SW) is converted to EWH in millimeters (mm) using the equation below:

$$SW = \frac{SW_{AF} \times q_2}{A} \times 10^6 \quad \text{(S2)}$$

where, q_2 is a conversion factor, $1.23346 \text{x} 10^{-6}$, for acre-feet to km^3, and A is the area of the study region, equivalent to 153,659 km^2.

The snow water equivalent from SNODAS is recorded as EWH in meters and can be directly converted into EWH in mm. The three surface water components were extracted for the study region and the three sub-basins, and subtracting their historical means (2003-2021) to obtain their anomalies in time series (Fig. 2(D) and Fig. S1(B)-(D)), and used to calculate groundwater anomalies using Equation 1.

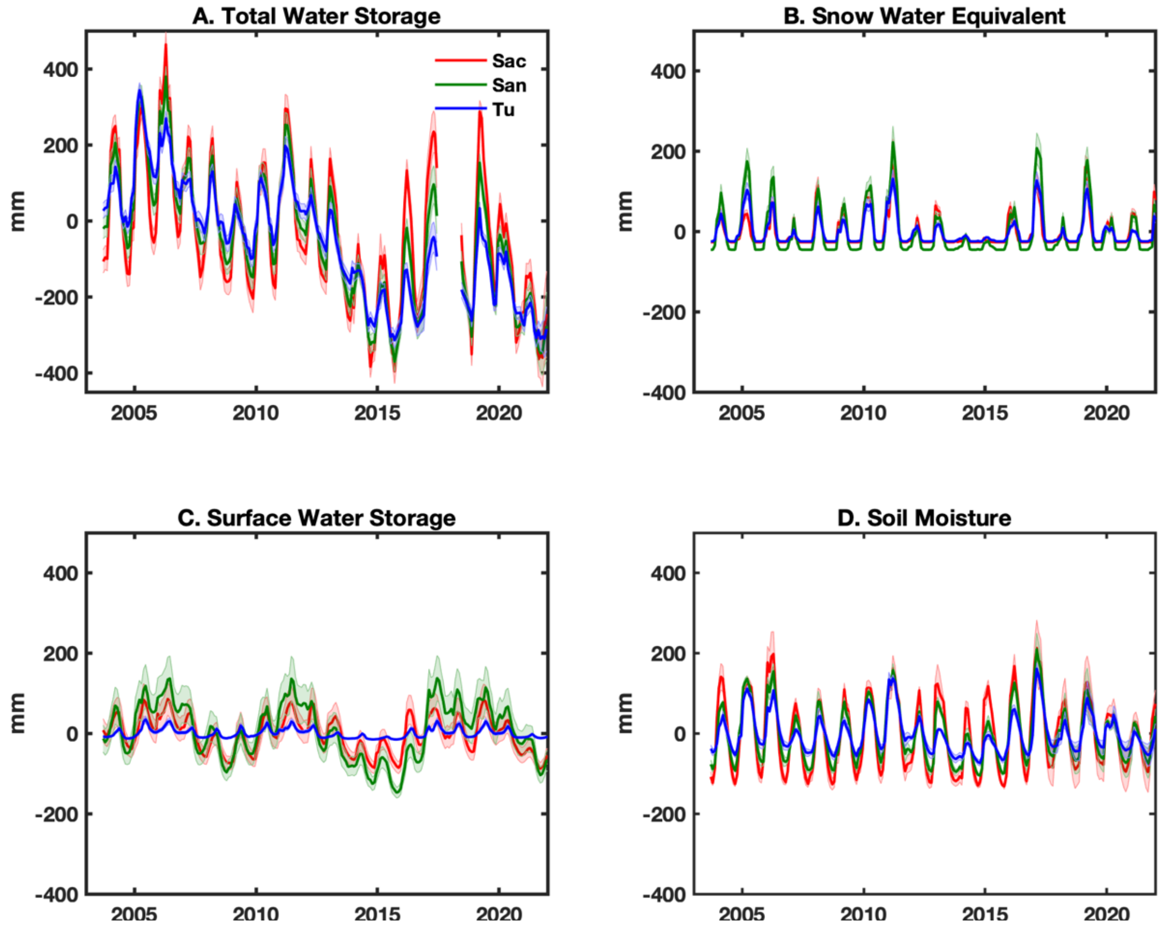

Fig. S1. Water storages components in the three sub-basins of the study region. (A) GRACE/FO derived total water storage anomalies. (B) Snow water equivalent (SWE) anomalies. (C) Surface water storage anomalies (SW). (D) Soil moisture anomalies (SM). Sac, San, and Tu represent Sacramento, San Joaquin, and Tulare basins, respectively.

S3. Uncertainty of groundwater recharge and depletion rates

The groundwater recharge or depletion rates (*b*) were calculated using linear regression with the linear equation as below:

$$y = bx + c \text{ (S3.1)}$$

where *y* is the groundwater anomaly, *x* is the time step in years, and *c* is the intercept of the linear equation. We quantified the uncertainty of groundwater change rates (Δb) by using student's *t* test.

$$\Delta b = t \times SE_b \text{ (S3.2)}$$

where, *t* is the *t* score from statistic t table which depends on sample numbers and the definition of the confidence interval (95% in the study), and SE_b is the standard error of the coefficient estimate.

$$SE_b = \frac{\sqrt{\frac{\sum_{i=1}^{n}\left(y_i-(\hat{b}x_i+\hat{c})\right)^2}{DR}}}{\sqrt{\sum_{i=1}^{n}(x_i-\bar{x})^2}} \quad \text{(S3.3)}$$

where, $\hat{b}$ and $\hat{c}$ are the slope (estimated groundwater change rate) and intercept from the regression, respectively; y_i and x_i are the groundwater anomalies at time x of sample i during the regression period; n is the total sample number for the regression, and *DR* is the degree of freedom, equivalent to n-2 for linear regression.

S4. Water table depth from wells

The *in situ* well measurements used in the study are identical to those used in Kim et al., (2021) (*1*), which were compiled and processed using groundwater monitoring networks managed by California's Department of Water Resources (DWR) and the U.S. Geological Survey (USGS). The archived raw data are inconsistent in well identification system, format, recording period and temporal resolution, and quality etc., which presents a challenge to integrate and merge the datasets for regional domain monitoring. Kim et al., (2021) (*1*) therefore developed a series processing scheme for data integration into a large domain. First, a filtering process was conducted based on Quality Control criteria provided from each management agency to remove erroneous measurements of each well. Then, these qualified measurements from all wells were merged and assigned a new identifier.

Because coordinates of wells from raw data do not have sufficient resolutions, some wells share the same or very close coordinates but with inconsistent values and nomenclature. To overcome this issue and only select dependable data, Kim et al., 2021 (*1*) applied a gridding and scoring system to only select high quality, unique data within each grid cell. The Central Valley domain was gridded into 1 km cells. All wells fell within an individual grid cell were scored based on 1. total number of measurements, 2. time coverage during the study period, and 3. mean measurement number per month. These criteria were normalized and weighted evenly to all wells. Measurements from the highest ranking well was then selected to represent the water table depth for that grid cell. Figure S2 shows the well stations distributed in the study region. The monthly water table depth of Central Valley and each basin domain were calculated by averaging the selected data across the domain, as shown in Fig. S3. Note that the well measurements from Sacramento basin may dominate water table depth variations for Central Valley due to about 4-6 times more samples as compared to the other two basins. Sample numbers for the entire Central Valley decrease by about 70% after 2019, which may result in an inconsistency in the time series of data, and may cause the discrepancy between well measurements and GRACE/FO estimates as shown in Fig. 3(B) in the main text.

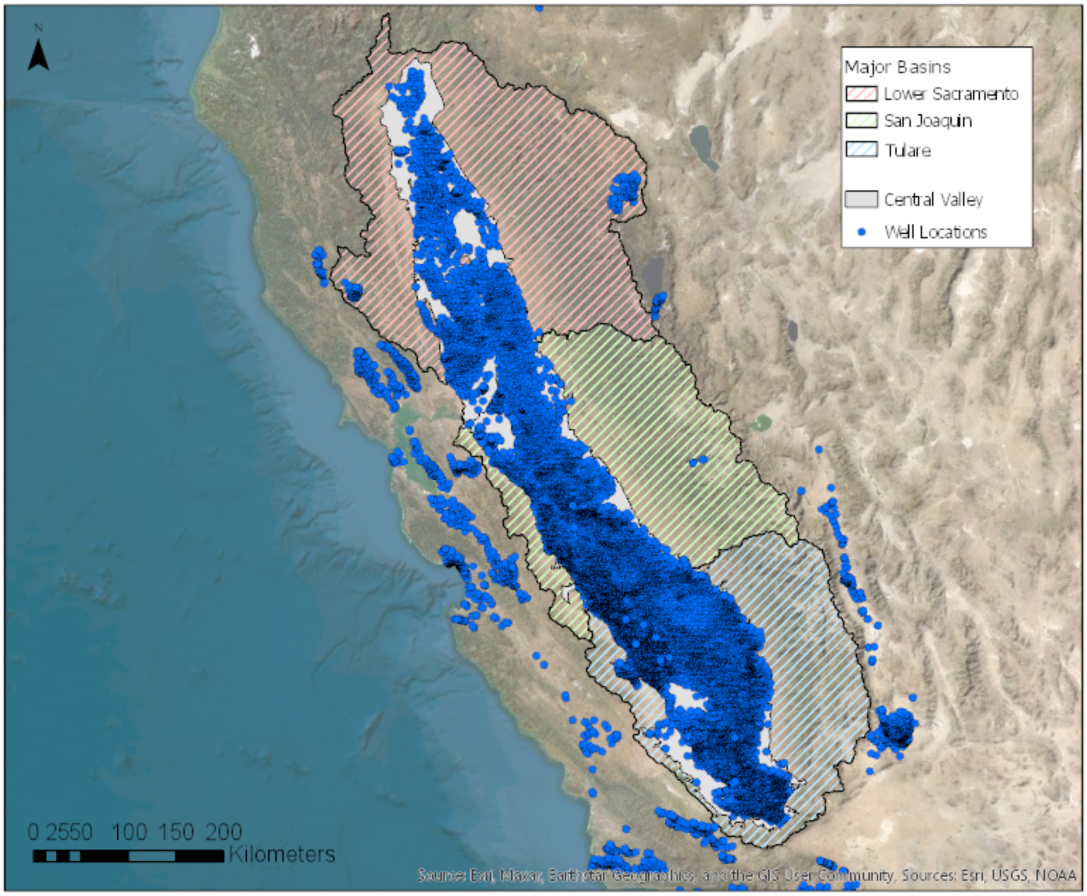

Fig. S2. Geographical locations of monitoring wells in the Central Valley. Well data falling within the Central Valley are used for this study.

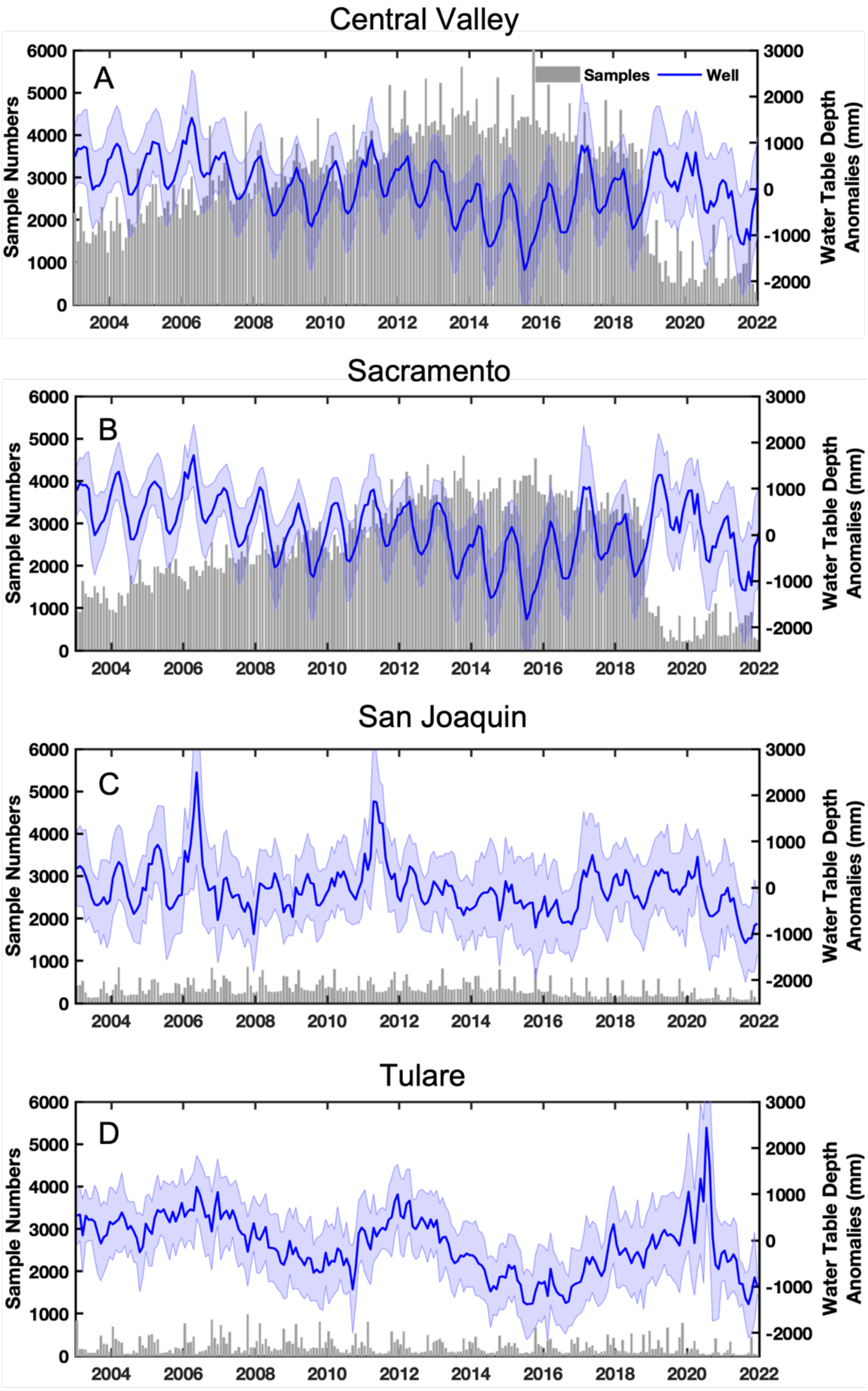

Fig. S3. Monthly water table depth anomalies and sample numbers of *in situ* well measurements. A. Water table depth anomalies for the Central Valley. B. Sacramento Basin. C. San Juaquin Basin. Grey bars represent sample numbers for the month, and the blue shades are the uncertainty of water table depth from standard deviation of measurements.

S5. Deseasonalized groundwater and water table depth anomalies

Observed water table depth anomalies (WTDA) in the Central Valley (Fig. S4(A)) are deseasonalized to remove their seasonal variations. The climatology over the study period (2003-2022) is constructed by averaging WTDA at a monthly scale and then plotted for the time period 2003-2022 (Fig. S4(B)). Deseasonalized WTDA is then obtained by subtracting the climatology from the WTDA (Fig. S4(C)). The same process is applied to GRACE/FO derived groundwater anomalies (Fig. S5). The seasonal variations are removed for the GRACE/FO and well data comparison to avoid the potential phase shift between the two datasets as shown in Results section and Fig. 3. The same comparisons are also conducted for the three sub-basins in Fig. 6.

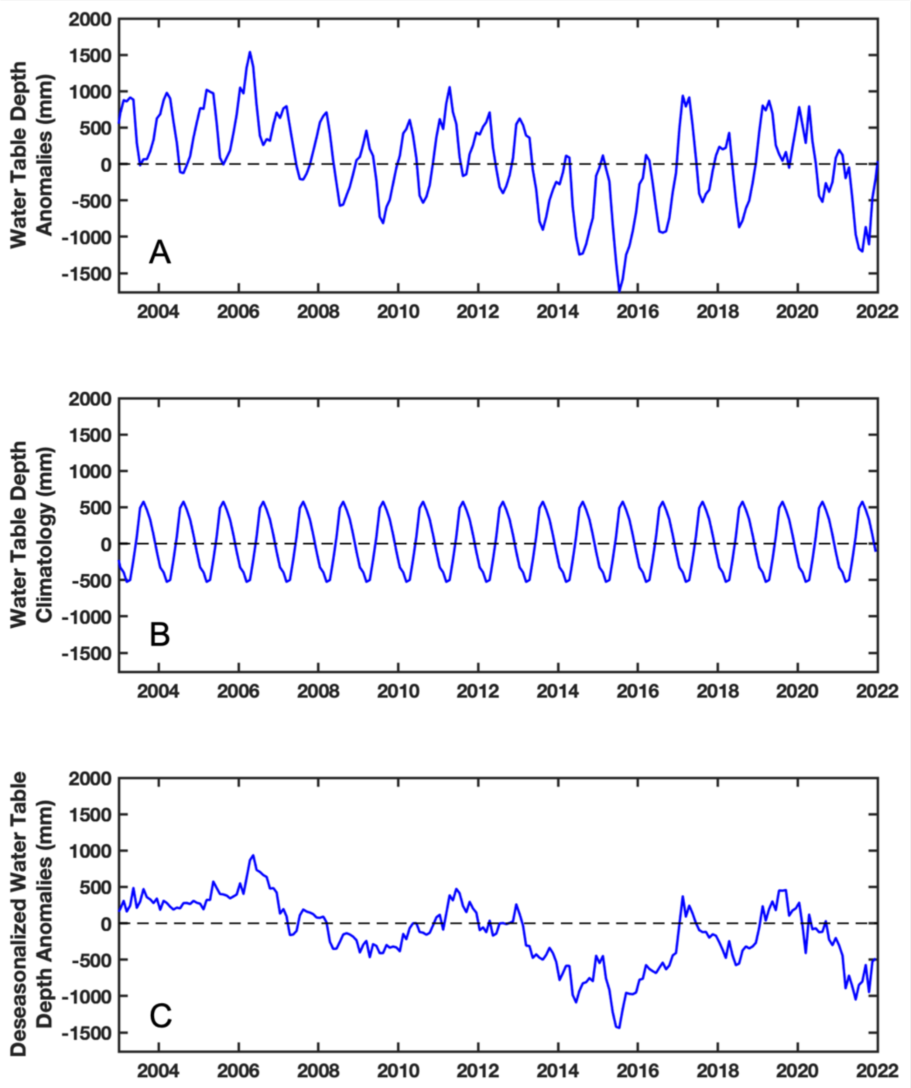

Fig. S4. Deseasonalized water table depth processing. A. Water table depth anomalies from wells for the Central Valley. B. Monthly climatology of water table depth anomalies. C. Deseaonalized water table depth anomalies for the Central Valley.

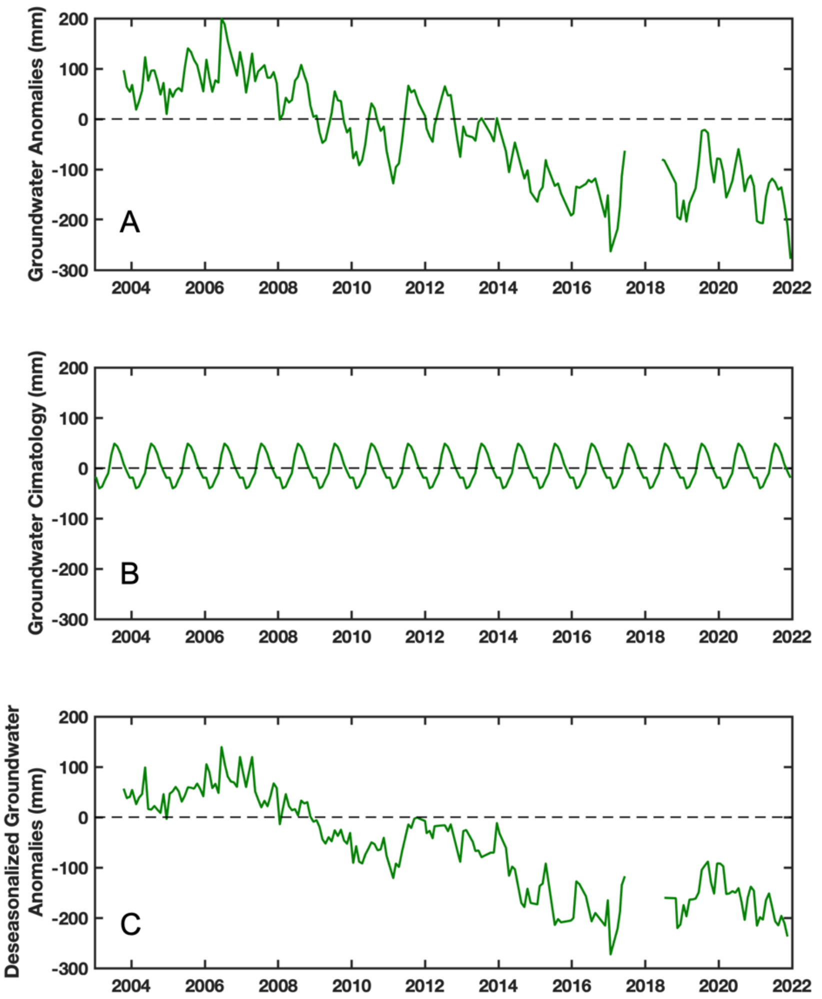

Fig. S5. Deseasonalized GRACE/FO groundwater anomalies processing. A. GRACE/FO groundwater anomalies for the Central Valley. B. Monthly climatology of groundwater anomalies. C. Deseaonalized groundwater anomalies for the Central Valley.

Reference:

1. Kim, K. H., Liu, Z., Rodell, M., Beaudoing, H., Massoud, E., Kitchens, J., Dudek, M., Saylor, P., Corcoran, F., & Reager, J. T. An evaluation of remotely sensed and in situ data sufficiency for SGMA-scale groundwater studies in the Central Valley, California. *J. Am. Water Resour. Assoc.*, 1-11, doi:10.1111/1752-1688.12898 (2021).
